# YKL-40/CHI3L1 facilitates migration and invasion in HER2 overexpressing breast epithelial progenitor cells and generates a niche for capillary-like network formation

**DOI:** 10.1007/s11626-019-00403-x

**Published:** 2019-09-03

**Authors:** Erika Morera, Sarah Sophie Steinhäuser, Zuzana Budkova, Saevar Ingthorsson, Jennifer Kricker, Aileen Krueger, Gunnhildur Asta Traustadottir, Thorarinn Gudjonsson

**Affiliations:** 1 Stem Cell Research Unit, Biomedical Center, Department of Anatomy, Faculty of Medicine, School of Health Sciences, University of Iceland, Vatnsmyrarvegi 16, 101 Reykjavik, Iceland; 2 Department of Laboratory Hematology, Landspitali - University Hospital, Reykjavik, Iceland; 3 Heidelberg Institute for Stem Cell Technology and Experimental Medicine, Heidelberg, Germany

**Keywords:** YKL-40/CHI3L1, Epithelial to mesenchymal transition (EMT), Migration, Invasive breast cancer, Angiogenesis

## Abstract

Epithelial to mesenchymal transition (EMT) is a developmental event that is hijacked in some diseases such as fibrosis and cancer. In cancer, EMT has been linked to increased invasion and metastasis and is generally associated with a poor prognosis. In this study, we have compared phenotypic and functional differences between two isogenic cell lines with an EMT profile: D492M and D492HER2 that are both derived from D492, a breast epithelial cell line with stem cell properties. D492M is non-tumorigenic while D492HER2 is tumorigenic. Thus, the aim of this study was to analyze the expression profile of these cell lines, identify potential oncogenes, and evaluate their effects on cellular phenotype. We performed transcriptome and secretome analyses of D492M and D492HER2 and verified expression of selected genes at the RNA and protein level. One candidate, YKL-40 (also known as CHI3L1), was selected for further studies due to its differential expression between D492M and D492HER2, being considerably higher in D492HER2. YKL-40 has been linked to chronic inflammation diseases and cancer, yet its function is not fully understood. Knock-down experiments of YKL-40 in D492HER2 resulted in reduced migration and invasion as well as reduced ability to induce angiogenesis in an in vitro assay, plus changes in the EMT-phenotype. In summary, our data suggest that YKL-40 may provide D492HER2 with increased aggressiveness, supporting cancer progression and facilitating angiogenesis.

**Electronic supplementary material:**

The online version of this article (10.1007/s11626-019-00403-x) contains supplementary material, which is available to authorized users.

## Introduction

Epithelial to mesenchymal transition (EMT) is a developmental process that describes the plasticity of epithelial cells to change phenotype from compact adherent epithelium to mesenchymal-like cells that have lost their polarity and cell-cell adherence (Thiery *et al*. 2009). This is an important event in normal development, during gastrulation, neural crest formation, and wound healing, but also takes place in malignant processes like fibrosis and cancer (Thiery *et al*. 2009; Nieto 2013). In cancer, EMT is associated with increased aggressiveness and it is believed to be necessary for migration and invasion of cancer cells for the initial steps in the metastatic processes (Thiery *et al*. 2009).

The EMT process is accompanied by increased expression of mesenchymal markers such as vimentin, N-cadherin, α-smooth muscle actin, fibronectin, and/or Axl, as well as decreased or absent expression of epithelial markers like cytokeratins, yet the most referenced change in EMT is the loss of E-cadherin (Peinado *et al*. 2007; Gjerdrum *et al*. 2010). EMT is known to be induced by a number of factors. It can take place due to intrinsic factors such as genetic mutations or epigenetic changes or extrinsic factors like hypoxia or inflammation that stimulate the release of signals from the stroma (Yang *et al*. 2008; Kalluri and Weinberg 2009; Polyak and Weinberg 2009). Transforming growth factor beta (TGF-β) has been shown to be a powerful inducer of EMT in a number of cell types (Kasai *et al*. 2005; Willis and Borok 2007; Kalluri and Weinberg 2009). Tyrosine kinase receptors and their ligands like EGF, PDGF, FGF, and HGF have also been shown to induce EMT (Kalluri and Weinberg 2009). Recently, regulatory functions carried out by non-coding RNAs have attracted the attention of researchers. Downregulation of miRNAs, especially members of the miR-200 family, miR-203, and miR-205, have been linked to EMT (Wiklund *et al*. 2010; Moes *et al*. 2012; DeCastro *et al*. 2013; Hilmarsdottir *et al*. 2014, 2015). In epithelial cells, the expression of these miRs is high but is commonly reduced or absent during EMT. Interestingly, rescue of these miRs can revert the EMT phenotype in the process mesenchymal to epithelial transition (MET) (Burk *et al*. 2008; Korpal *et al*. 2008; Hilmarsdottir *et al*. 2014). Furthermore, EMT in cells is often accompanied by increased ability to migrate and invade, as well as increased resistance to apoptosis (Thiery *et al*. 2009). For this reason, EMT has been related to aggressiveness and metastasis in cancer (Thiery 2002; Nieto 2013; Tan *et al*. 2014). The concept of cancer stem cells (CSCs) has also been linked to EMT, where EMT is an integral part of CSC plasticity and survival (Mani *et al*. 2008). Like somatic stem cells, CSCs have the capability to self-renew and their EMT phenotype is believed to be protective against external damages, which could lead to resistance to cancer therapies (Al-Hajj *et al*. 2003; Stingl 2006). In addition, CSCs as a cell population could be the cells responsible for the dissemination of cancer cells and the heterogeneity evidenced in metastasis (Brabletz 2012). Therefore, it is vital to elucidate the role of CSCs in cancer progression in order to improve efficient treatments and targeted therapy.

In breast cancer, the subtype basal-like breast cancer (BLBC) is identified as having the most undifferentiated cells and heterogeneity, which has been linked to CSCs and interestingly has a higher incidence of EMT (Mani *et al*. 2008; Morel *et al*. 2008; Sarrio *et al*. 2008). However, EMT is not exclusively found in this subtype. In luminal and HER2 breast cancers, there is an EMT-derived phenotype, although at a lower percentage (Yu *et al*. 2013; Tan *et al*. 2014).

Resistance to drugs in breast cancer may be associated with EMT-phenotype and heterogeneity of cells in the tumor. It is important to note that states of EMT are not merely defined as completely epithelial or mesenchymal; there are intermediate states of EMT that provide plasticity and advantages to cells to adapt to their microenvironment. This program is referred as partial-EMT (p-EMT) (Huang *et al*. 2013; Nieto 2013; Tam and Weinberg 2013; Yu *et al*. 2013).

The epithelial cells that reside in the breast gland are under continuous remodeling due to hormonal cycling and interactions with the surrounding stroma. This crosstalk is required in normal development and differentiation, and is utilized by tumor cells during cancer progression. Resident fibroblasts and macrophages in the stroma are able to produce growth factors and pro-inflammatory molecules, but also pro-angiogenic molecules that support the creation of new blood vessels. Fibroblasts not only interact with epithelial cells, they also activate the action of immune cells like macrophages and promote angiogenesis by cell communication with endothelial cells. The vascular endothelial growth factor (VEGF) and TGF-β are secreted by fibroblasts and macrophages, and they are able to stimulate angiogenesis. TGF-β induces the expression of VEGF that is secreted to the ECM and stimulates endothelial cells to initiate angiogenesis (Relf *et al*. 1997; Carmeliet and Jain 2000).

There is also direct interaction between epithelial cells and endothelial cells. The vascular niche plays an important role in transporting oxygen and nutrients and releasing signals for its correct morphogenesis and development. Indeed, it has also been observed that endothelial cells increase growth and branching morphogenesis of breast epithelium (Shekhar *et al*. 2000; Sigurdsson *et al*. 2006; Ingthorsson *et al*. 2010) and induce EMT (Sigurdsson *et al*. 2011). In the context of cancer, the high proliferation rate of tumor cells can lead to a reduction in oxygen concentration resulting in acidification of the surrounding stroma. In order to survive, cancer cells are able to secrete signals that induce angiogenesis, such as VEGF (Hanahan and Weinberg 2011). Receptors on endothelial cells, such as VEGFR2, are induced to trigger activation of the transcriptional machinery of angiogenesis that involves the hypoxic inducible factor 1 (HIF1) and other factors like nuclear factor κB (NF-κB) (Vegran *et al*. 2011; Sonveaux *et al*. 2012). The increase in angiogenesis ensures oxygen and a nutrient supply and therefore results in a worse prognosis of the cancer, making it an important factor to study.

D492 is a breast progenitor epithelial cell line that was established by immortalization of a suprabasal subpopulation of breast tissue from a healthy donor (Gudjonsson *et al*. 2002). As reviewed in Briem *et al*. (2019), D492 can generate both luminal and myoepithelial cells, and in 3D culture forms branching terminal ductal lobular units (TDLU) like structures (Briem *et al*. 2019). We have previously demonstrated that when D492 is co-cultured with breast endothelial cells (BRENCs), a subpopulation of cells undergoes EMT. One such subpopulation was isolated, giving rise to the D492M cell line, which has a fixed mesenchymal phenotype (Sigurdsson *et al*. 2011). In addition, we have shown that D492 cells with forced overexpression of the HER2 oncogene (D492HER2) have lost their epithelial phenotype and gained a mesenchymal one (Ingthorsson *et al*. 2015).

In this study, we have compared functional and phenotypic differences between D492M and D492HER2. We show here that D492HER2 proliferates, migrates, and invades faster than D492M. Furthermore, glucose metabolism is more dependent on glycolysis than oxidative phosphorylation in D492HER2. The comparative analysis of transcriptome revealed that a glycoprotein, YKL-40, also known as CHI3L1, is highly upregulated in D492HER2 and may contribute to the differences in tumorigenicity between the cell lines. YKL-40 has previously been linked to chronic inflammation diseases and cancer (Libreros *et al*. 2012; Jefri *et al*. 2015; Libreros and Iragavarapu-Charyulu 2015; Cohen *et al*. 2017); however, its function is not clearly understood yet. Functional studies using knockdown of YKL-40, recombinant protein, and overexpression experiments reveal that YKL-40 influences migration, invasion, and angiogenesis and induces changes in the EMT phenotype.

## Material and Methods

### Cell culture (2D and 3D)

The main cell lines used in this project were D492 (Gudjonsson *et al*. 2002) and its EMT-phenotype-derived cell sublines D492M (Sigurdsson *et al*. 2011) and D492HER2 (Ingthorsson *et al*. 2015). For monolayer culture, flasks or plates were pre-coated with collagen I (2.2%) (no. 5005-B, Advanced BioMatrix, Carlsbad, CA). The medium used for culturing the cell lines was H14 (Blaschke *et al*. 1994), an enriched serum-free medium based on DMEM:F12 in which growth factors are added (insulin, transferrin, EGF, sodium selenite (NaSel), estradiol, hydrocortisone, prolactin).

Human umbilical vein endothelial cells (HUVECs) were isolated from umbilical cords obtained from Landspitali University Hospital, Reykjavik, Iceland, with informed consent and approved by the Landspitali ethical committee (No. 35/2013). HUVECs were cultured in EGM2 + 5% FBS (no. CC-3162, Lonza).

In 3D cultures, cells were cultured in 300 μL of Matrigel (no. 354230, Corning, Corning, NY) per well, in 24-well plates (no. 353047, Corning, Corning, NY). In monoculture, 20,000 cells were embedded in reconstituted basement membrane, rBM, purchased as Matrigel (Corning no. 354230), with 500 μL of H14 media on top, while in co-cultures, 500 cells of D492 or its sublines were co-cultured with 150,000–200,000 HUVECs, embedded in Matrigel, with 500 μL of EGM2 + 5% FBS media on top.

### Gene expression levels by qRT-PCR

Total RNA was isolated with cold Tri-Reagent reagent (no. AM9738, Life Technologies, Carlsbad, CA). RNA precipitation was done using isopropanol and centrifugation at 14,000 rpm for 20 min. Afterwards, RNA was washed with ethanol two times. The RNA pellet was diluted in RNAse free water. Concentration of RNA was measured using a NanoDrop® ND-1000 UV/Vis-Spectrophotometer (Thermo Fisher Scientific, Waltham, MA). For cDNA synthesis, SuperScript IV (no. 18090-200, Thermo Fisher Scientific) and random hexamer primers were used.

To quantify the expression level of genes, TaqMan (no. M3004L, NEB) or SYBR Green (no. M3003L, NEB) chemistries were used to detect gene expression. Comparative Ct values were determined using an ABI 7500 instrument (Applied Biosystems, Foster City, CA). All used primers are listed in Table 1. GAPDH was used as the reference gene.

**Table 1.** List of primers used in this study. List of gene IDs for all target genes used and their corresponding primer IDs and supplier

Primers	Cat. number	Company
miR-200c	No. YP00204482	Qiagen
miR-203	No. 205914	Exiqon
miR-205	No. 204487	Exiqon
VEGF-A	Hs.PT.58.21234833	IDT
VEGF-C	Hs.PT.58.14602240	IDT
GUCA1C	Hs.PT.58.680712	IDT
CITED1	Hs.PT.58.1567731	IDT
MYBPH	Hs.PT.58.39772389	IDT
YKL-40 /CHI3L1	Hs.PT.58.22570467	IDT
KCNQ1OT1	Hs.PT.58.4572396.g	IDT
ERBB2	Hs.PT.58.1330269	IDT
GPR27	Hs.PT.58.38722549.g	IDT
S100A9	Hs.PT.58.20989743	IDT
DLK1	Hs.PT.58.40622309	IDT
GDF6	Hs.PT.58.20193545	IDT
PXDN	Hs.PT.58.630748	IDT
TLR4	Hs.PT.58.38700156.g	IDT
BMP4	Hs.PT.56a.3848863	IDT
CDH2	Hs.PT.58.26024443	IDT

### Expression levels of miRNAs by qRT-PCR

Total RNA was extracted with Tri-Reagent (no. AM9738, Thermo Fisher Scientific). The RNA was reverse transcribed using miRCURY LNA RT Kit (no. 339340, Qiagen, Hilden, Germany) for cDNA synthesis reactions. Quantitative RT-PCR analysis of miRNAs was performed using miRCURY LNA SYBR Green PCR Kit (no. 339346, Qiagen). Relative expression was calculated with the 2^∆∆Ct^ method. All used primers are listed in Table 1. Normalization was done with U6 (no. 203907, Exiqon, Vedbaek, Denmark).

### Protein levels by Western Blot (WB)

Protein isolation was done using RIPA buffer, and the concentration was measured by the colorimetric Bradford method. Equal amount of protein was loaded for each sample (5 μg) in precast NuPAGE 10% Bis-Tris gels (Invitrogen, Carlsbad, CA). Proteins were detected using IRDye secondary antibodies on an Odyssey imaging system (Li-Cor Biosciences, Lincoln, NE). Loading controls actin or tubulin were used for quantification. Antibodies used for WB are listed in Table 2.

**Table 2.** List of antibodies used in this study. List of primary antibodies (protein IDs, dilution, company, and order IDs) for western blotting and IF staining for candidate proteins

Antibodies	Assay	Cat. number	Company	Dilution
Actin	WB	ab3280	Abcam	1:5000
Axl	IF	CS no. 8661	Cell Signaling	1:100
CK14	WB	ab15461	Abcam	1:1000
CK19	IF	ab7754	Abcam	1:100
E-cadherin	WB	610182	BD Transduction Labs	1:1000
Tubulin	WB	ab6046	Abcam	1:5000
Vimentin	WB	M0725	DAKO	1:1000
YKL-40 /CHI3L1	IF	MABC196	Millipore	1:100
YKL-40 /CHI3L1	WB	MABC196	Millipore	1:500

### Immunostaining

Immunofluorescence (IF) was used to detect proteins and visualize their subcellular location. Cells were fixed with 3.7% paraformaldehyde (PFA) and permeabilized with 0.1% Triton X-100. Blocking with FBS was done prior to incubation with primary antibodies. Incubation was done overnight at 4°C and followed by incubation of secondary antibodies conjugated to fluorochromes Alexa Fluor-488, Fluor-546, or Fluor-647 for 1 h at room temperature. For nuclei staining, DAPI was used. Imaging was done using an EVOS FL Auto 2 Cell Imaging System (Thermo Fisher Scientific) or FV1200 Olympus inverted confocal microscope. Antibodies used for IF are listed in Table 2.

### Transcriptome data analysis and classification

Total RNA sequence profiles for D492, D492M, and D492HER2 were obtained from normal cell cultures and treated following the protocol detailed in Halldorsson *et al*. (2017).

Differentially expressed genes between cell lines were further classified into categories to see the biological processes that were enriched. Using a differential range from two fold change, genes were analyzed by the PANTHER analysis database (www.pantherdb.org) following instructions of Mi *et al*. (2013).

### Secretome data analysis

For mass spectrometry, D492 lines were grown in T175 flasks (no. 353112, Corning) and conditioned medium (CM) was collected, concentrated for 55 min using EMD Millipore Amicon™ Ultra-15 Centrifugal Filter Units (no. UFC900324, Merck Millipore, Darmstadt, Germany) followed by buffer exchange to 100 mM TRIS/HCl buffer. Triplicate samples were stored at − 80°C. Label-free relative protein quantification by nLC MS/MS after trypsin digestion was performed at the FingerPrints Proteomics Facility, University of Dundee, Dundee, UK and raw data were analyzed using MaxQuant software (version 1.6.2.1). Quantitative and statistical analyses were performed using XLStat (version 2018.1). Data were *P* value corrected (significance level 0.05) and sorted based on ≥ 2-fold higher secretion (LFQ intensity) by D492HER2 compared to D492M.

### Migration and invasion assays

Migration and invasion assays were done in 24-well plates with transwell filter inserts (no. 353097, Corning) of 8 μm size pore diameter. Transwell inserts in the migration assay were pre-coated with collagen I (2.2%) and in the invasion assay; they were pre-coated with Matrigel diluted 1:10 in H14 media. Fifty thousand cells/transwell were seeded on the upper chamber in H14 media. In the bottom chamber, H14 was supplemented with 10% FBS as a chemoattractant. A cotton swab was used to remove non-migrated and non-invaded cells after 24 h and after 48 h, respectively. Thereafter, cells were fixed with 3.7% PFA and stained with crystal violet (10%) or DAPI (1:5000 dilution) for 30 min. Three random pictures were taken per well and the number of cells was quantified. For DAPI-stained samples, images were converted to 8-bit in ImageJ (version 2.0.0), threshold-adjusted, and binary-converted and migratory/invasive cells were counted using the *analyze particles* function.

### Proliferation assay

Proliferation of cells was determined by seeding 10,000 cells/well in triplicate in 24-well plates in H14 (D492 cell lines) or EGM5 (HUVECS). Every day (2 d for HUVECs), cells were fixed and stained with crystal violet (10%). Crystal violet was diluted with acetic acid and the OD was measured at 570 nm wavelength. Alternatively, cell viability was assessed using PrestoBlue™ Cell Viability Reagent (ThermoFisher Scientific, Waltham, MA). Cells were seeded in H14 media in a 96-well plate at a density of 3000 cells/well and cultured for 4 d. PrestoBlue was added (1/10th of the total volume) to each well and incubated for 4 h, and absorbance was read on a plate reader at 570 nm and 595 nm.

### Apoptosis assay

To quantify apoptosis, cleavage of caspase 3/7 was measured by a luciferase assay (ApoTox-GloTM Triplex Assay, Promega, Madison, WI). Apoptosis was induced by incubating cells with 10 μM camptothecin (CPT) for 24 h according to the manufacturer’s protocol. After cellular lysis, luciferase was measured with a microplate reader ModulusTM II (Turner Biosystems, Sunnyvale, CA).

### Glucose consumption and lactate production measurements

Glucose uptake was measured using Glucose Uptake-GloTM kit (no. J1341, Promega) following the manufacturer’s protocol. Briefly, the analogue of glucose, 2-deoxyglucose (2DG), was added to the media and taken up by cells. When transported into cells, 2DG is phosphorylated to 2-deoxyglucose 6-phosphate (2DG6P) and further metabolization stimulates luciferase reactions and luminescence was measured by the microplate reader Modulus TM II (Turner Biosystems, Sunnyvale, CA).

Glucose consumption and lactate production were measured from the collected media when cells were in a high confluency. Metabolites were measured at the Analyzer machine (ABL90 FLEX Analyzer, Radiometer) at the Blood Bank of Landspitali (Reykjavik, Iceland).

### Neutralization assay of YKL-40 protein

A monoclonal antibody against YKL-40 (mA^YKL40^) (MABC196, Millipore) was used to block the secretion of YKL-40 in D492HER2. The antibody was diluted in fresh H14 medium at a concentration of 10 μg/mL. Medium from cells incubated for 24 h with mA^YKL40^ was collected, and medium from non-treated D492HER2 cells was used as control. Conditioned media (CM) were used for tube formation assays (described below).

### Tube formation assay on endothelial cells (angiogenesis assay in vitro)

To simulate angiogenesis in vitro, 10,000–12,000 HUVECs were seeded on top of 10 μL solidified rBM in a 96-well angiogenesis plate (no. 89646, Ibidi). Controls included HUVECs cultured in EGM5 media and a dilution of 1:1 EGM5 and conditioned media (CM). Recombinant YKL-40 protein (YKL-40^r^) (no. 11227H08H5, Thermo Fisher Scientific, Waltham, MA) was added to the medium at a final concentration of 100 ng/mL. After incubation overnight, the endothelial network was imaged using the EVOS FL Auto 2 Cell Imaging System. Analysis and quantification were done using the *Angiogenesis analyzer* plug-in on ImageJ software (version 2.0.0).

### Transient knockdown of YKL-40 by siRNA

Pre-designed siRNAs (Silencer® Select Pre-Designed, Validated and Custom siRNA, Life Technologies) against YKL-40 were used at a concentration of 10 nM in D492HER2 cells to downregulate YKL-40. First, cells were seeded on 6-well plate (no. 353046, Corning, Corning, NY) and after 24 h, cells were transfected with the siRNA using chemical transfection with Lipofectamine® RNAiMAX (no. 13778150, Life Technologies, Carlsbad, CA). For each experiment, two different siRNAs against YKL-40 and a negative control siRNA (no. 4390843, Life Technologies, Carlsbad, CA) were used. The siRNAs are listed in Table 3. After 48 h, the knockdown was confirmed by qRT-PCR and by WB.

**Table 3.** siRNAs used for transient YKL-40 knockdown. Catalog numbers of siRNAs used as negative control and transient knock-down of YKL-40

siRNAs	Product name	Cat. number	Company
Neg Ctrl siRNA	Silencer® Select Negative Control No. 1 siRNA	4390843	ThermoFisher Scientific
YKL-40 siRNA 1	Silencer® Select CHI3L1 siRNA 1	AM16708 (ID 119124)	ThermoFisher Scientific
YKL-40 siRNA 2	Silencer® Select CHI3L1 siRNA 2	AM16708 (ID 119126)	ThermoFisher Scientific

### Generation of stable cell line with knockdown of YKL-40 in D492HER2 by CRISPR

Specific gRNAs to knockdown YKL-40 were designed with the publicly available online ATUM CRISPR tool (https://www.atum.bio/). Annealing of the forward and reverse gRNA oligos was followed by ligation with the backbone vector pMLM3636. High-efficiency competent bacteria (no. C2987H, NEB) were transformed, and confirmation of successful DNA insertion was done by colony PCR. Plasmids were sequenced to confirm correct gRNA sequences. Transfection of D492HER2 was done with four plasmid gRNAs and a plasmid with only a Cas9 cassette (pST1374) using Lipofectamine® 3000 (Thermo Fisher). The control cell line was generated by transfecting the cells with only the Cas9 cassette. Selection of cells with insertion of Cas9 was done using blasticidin. Knockdown of YKL-40 in D492HER2 was confirmed by qRT-PCR and WB. Subsequently, the cell line that gave the best efficiency of knockdown was selected for further work. The sequence of this gRNA is in Table 4. It should be noted that D492HER2 is a cell line not suitable for single-cell cloning; therefore, a pool of cells was used for confirmation of knockdown and further experiments.

**Table 4.** gRNAs used for generating stable cell lines. Names and sequences of gRNAs used to either knockdown or overexpress YKL-40

gRNAs	Sequence	Action	Company
YKL-40/CHI3L1 KD	CCGCCATTTCTGCGCACCCA	Knockdown	
YKL-40/CHI3L1 SAM OV 1	AGTTTTGAAAACTTTGGGTC	Overexpress	Genscript
YKL-40/CHI3L1 SAM OV 2	CTGCCAGCAGAAGAGCCACT	Overexpress	Genscript

### Generation of stable cell lines overexpressing YKL-40 in D492 and D492M by CRISPRa

Overexpression of YKL-40 in D492 and D492M was carried out using the modified CRISPR strategy, called CRISPRa (Zhang *et al*. 2015). Two specific SAM gRNAs for YKL-40 and one empty control were purchased from Genscript containing zeomycin resistance cassettes. The gRNA sequences are listed in Table 4. Transfection of HEK293T cells was done to produce viral particles including gRNAs. Viruses collected were used to infect D492 and D492M cells that were previously transfected with dCas9-VP64 to activate the promoter and later induce overexpression of the selected gene. Transduction with gRNAs was performed and selection was done with zeocin (no. R25005, Thermo Fisher Scientific). Confirmation of overexpression of YKL-40 in D492 and D492M was done by qRT-PCR, WB, and IF.

### Statistical analysis

One-way analysis of variance (ANOVA) using Dunnett’s multiple comparison test or unpaired *t* tests were performed using GraphPad Prism to test significance. *P* values below 0.05 were considered significant (*p ≤ 0.05, **p ≤ 0.01, ***p ≤ 0.001, ****p ≤ 0.0001).

## Results

### Phenotypic and functional characterization of D492M and D492HER2

**Figure 1.**
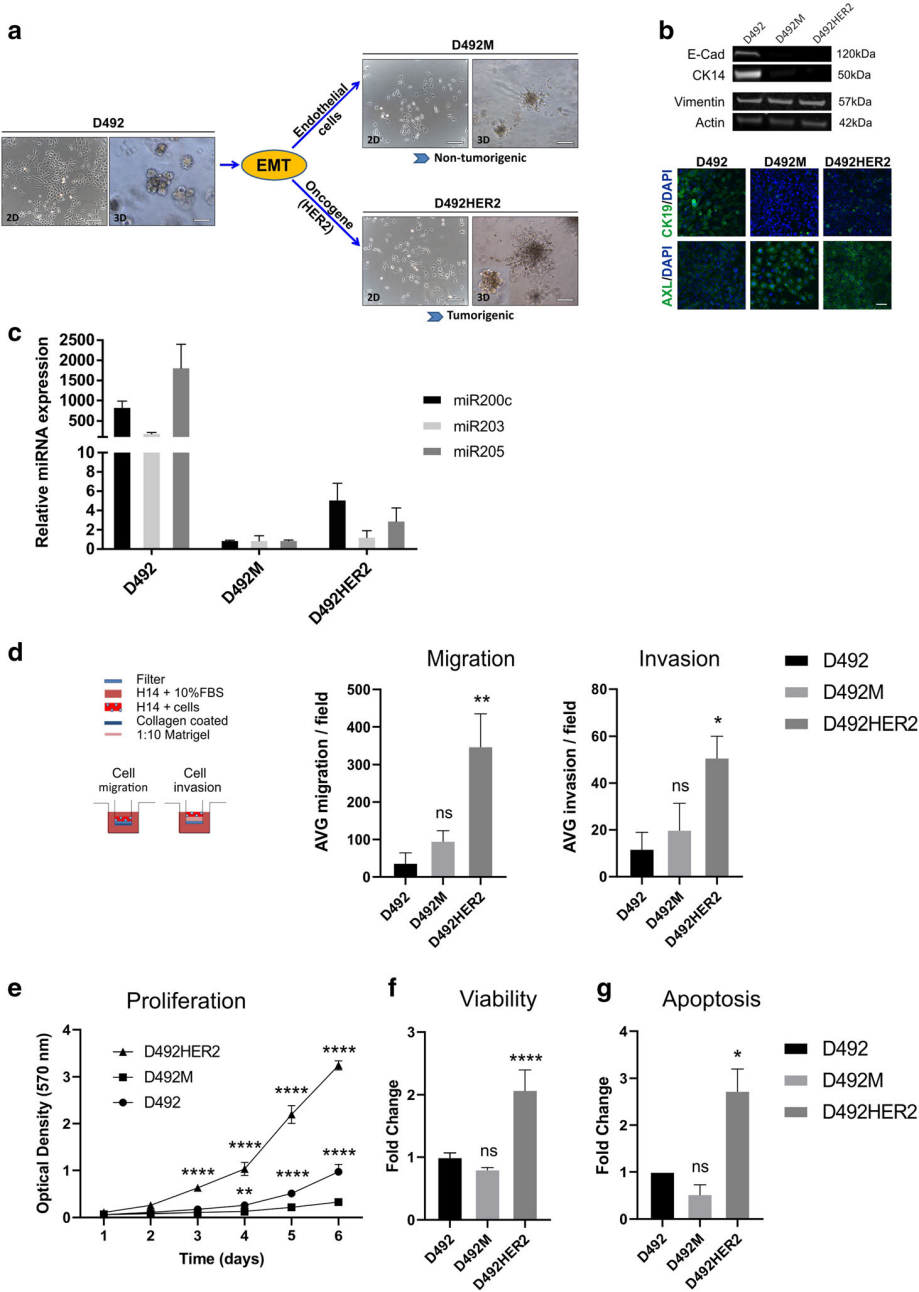
Phenotypic and functional characterization of D492M and D492HER2 cell lines. (*a*) D492M and D492HER2 are isogenic EMT-derived sublines of D492, a breast epithelial cell line with progenitor properties, which in 3D cell culture forms branching structures, resembling the TDLUs of the breast. D492M and D492HER2 were generated when D492 underwent endothelial-induced and oncogene (HER2)-induced EMT, respectively. D492M and D492HER2 show similar EMT-phenotype in 2D, but in 3D, D492M generate spindle-like colonies whereas D492HER2 generate spindle-like and grape-like colonies. D492M and D492HER2 also differ in their ability to generate tumors, as only D492HER2 is able to form tumors. *Scale bar* = 200 μm. (*b*) D492M and D492HER2 have lost expression of epithelial markers (E-cadherin, cytokeratin-14, and cytokeratin-19) and gained expression of mesenchymal markers (Axl and vimentin) as shown by western blotting and immunofluorescence. *Scale bar* = 100 μm. (*c*) D492M and D492HER2 show reduction in the expression of epithelial microRNAs such as miR-200c, miR-203, and miR-205 compared to D492. The lowest levels of expression are found in D492M (mean ± SD, *n* = 2). (*d*) In transwell migration and invasion assays, D492HER2 has an increased ability to migrate and invade compared to D492 and D492M. Results are shown as average number of cells per field (mean ± SEM, *n* = 3). One-way analysis of variance (ANOVA) and Dunnett’s multiple comparison test were used to test significance (*p ≤ 0.05, **p ≤ 0.01). (*e*) D492HER2 cells proliferate at a higher rate than D492 and D492M cells as shown by staining with crystal violet. Results are shown as average of four replicates (mean ± SD). Statistical significance was assessed using multiple *t* tests (one per *row*) (**p ≤ 0.01, ****p ≤ 0.0001). (*f*) Furthermore, increased proliferation rate of D492HER2 compared to D492 and D492M cells was demonstrated using PrestoBlue™ Cell Viability reagent. Results are shown as average of eight replicates normalized to D492 (mean ± SD). One-way analysis of variance (ANOVA) and Dunnett’s multiple comparison test were used to test significance (****p ≤ 0.0001). (*g*) In addition, D492HER2 cells are more susceptible to chemically induced apoptosis as compared to D492 and D492M cells. Caspase 3/7 luciferase activity was measured by luminescence and normalized to D492 (mean ± SEM, *n* = 2). Significance was assessed with one-way analysis of variance (ANOVA) and Dunnett’s multiple comparison test (*p ≤ 0.05).

D492M and D492HER2 are isogenic cell lines that share a partial-EMT phenotype but are different in terms of their ability to form tumors in mice (Sigurdsson *et al*. 2011; Ingthorsson *et al*. 2015). D492M and D492HER2 are both derived from D492, a breast epithelial progenitor cell line, through endothelial-induced EMT and oncogene-induced EMT, respectively (Fig. 1*a*). D492M and D492HER2 have lost the expression of epithelial markers like E-cadherin, CK14, and CK19 and gained expression of some mesenchymal markers like vimentin and Axl, (Fig. 1*b*). MicroRNAs (miRs) such as miR-200c, miR-203, and miR-205 are frequently downregulated in epithelial cells undergoing EMT, and this was the case for D492M and D492HER2. While D492 showed high expression of miR-200c, miR-203, and miR-205, these microRNAs were greatly reduced in D492HER2 and relatively absent in D492M (Fig. 1*c*). The difference in expression levels between D492M and D492HER2 may have been due to a more intermediate state of EMT for D492HER2. This intermediate state was also supported by the glucose metabolism (Fig. S1). These data show D492HER2 is more comparable in glucose uptake to D492 than D492M.

Further characterization demonstrated functional differences between the cell lines. Notably, D492HER2 cells migrated and invaded through transwell filters more efficiently than both D492M and D492 (Fig. 1*d*). The cell proliferation rate of D492HER2 cells was also significantly higher than in D492 and D492M cells (Fig. 1*e*, *f*). Furthermore, D492HER2 cells were more susceptible to chemically induced apoptosis than D492 and D492M cells (Fig. 1*g*), despite an apparent shift from oxidative phosphorylation to glycolysis as D492HER2 had higher glucose consumption and higher lactate production than either D492 or D492M cells (Fig. S1).

The functional differences between D492M and D492HER2 in terms of tumorigenicity prompted us to look into gene expression and secretome of D492M and D492HER2 to search for potential candidates responsible for these effects.

### Transcriptome and secretome analyses show that YKL-40 is enriched in D492HER2

**Figure 2.**
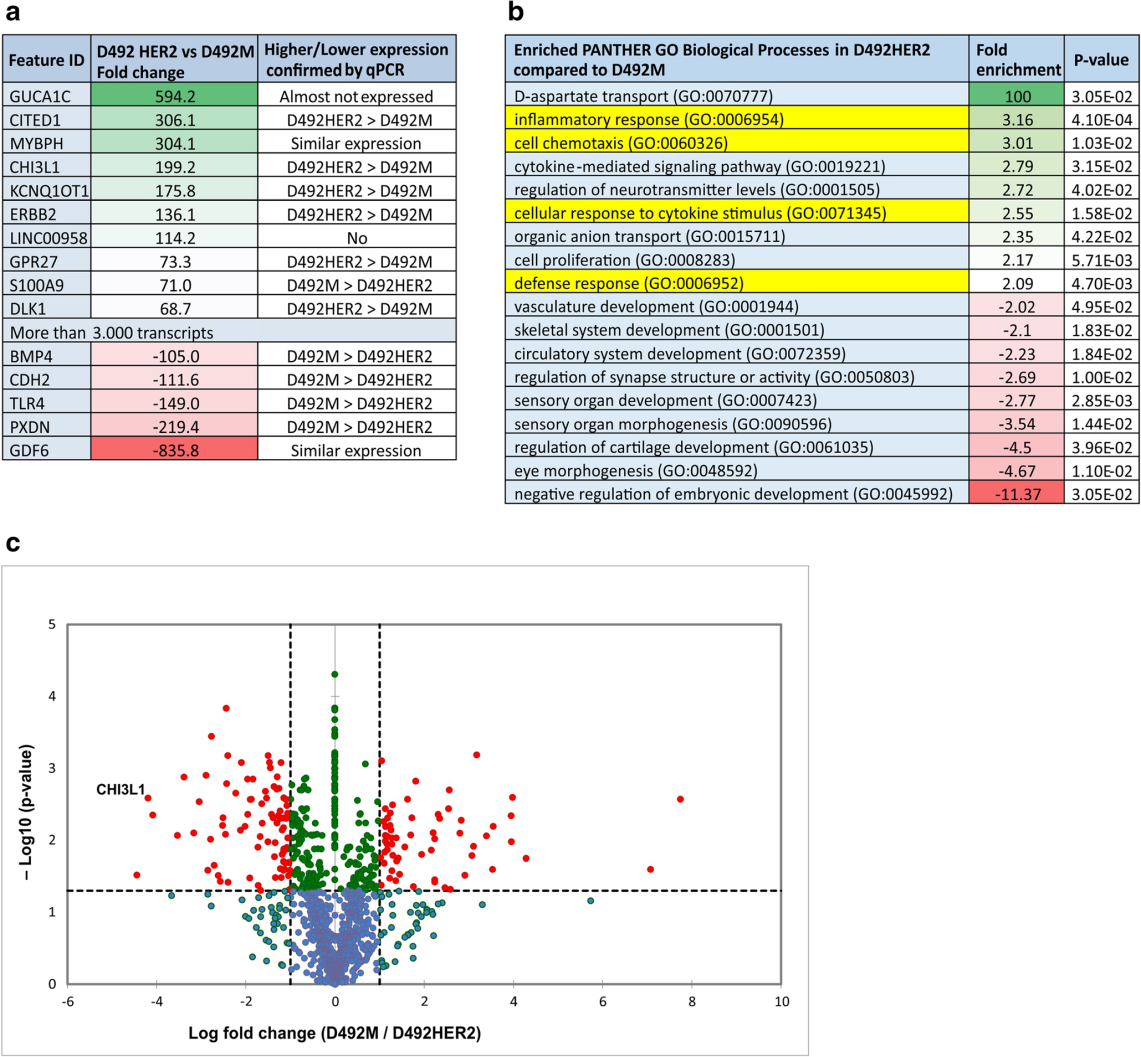
YKL-40 is enriched in D492HER2. *a* The analysis of transcriptome data comparing D492M and D492HER2 revealed more than 3000 transcripts differently expressed (more than two fold). YKL-40 (or CHI3L1) is more highly expressed in D492HER2 compared to D492M. *b* Classification of genes in biological processes shows an enrichment in D492HER2 compared to D492M in D-aspartate transport, inflammatory response, cell chemotaxis, cytokine-mediated signaling pathway, regulation of neurotransmitter levels, cellular response to cytokine stimulus, organic anion transport, cell proliferation, and defense response. YKL-40 is involved in the processes that require interaction with the stroma: inflammatory response, cell chemotaxis, cellular response to cytokine stimulus, and defense response (marked with *yellow*). *c* Mass spectrometry analysis of secreted proteins in CM revealed that YKL-40 is enriched in the secretome of D492HER2 compared to D492M (*p* value = 0.01).

Next, we employed genome-wide analysis to reveal differences in gene expression in D492M and D492HER2. More than 40,000 transcripts were differentially expressed between D492M and D492HER2 and the most 15 differentially regulated genes were confirmed by qPCR (Fig. 2*a*). As expected, HER2 (ErbB2) has much higher expression in D492HER2 cells due to the ectopic overexpression of the gene (Ingthorsson *et al*. 2015). Although CITED1 was the first validated candidate, its absolute level of expression was low; thus, we focused our interest on a gene very highly expressed in D492HER2 compared to D492M (and also to D492), namely YKL-40 (also known as CHI3L1). The function of YKL-40 is not clearly understood but it has been suggested to be involved in cancer progression and other inflammation diseases (Libreros *et al*. 2012; Libreros and Iragavarapu-Charyulu 2015; Cohen *et al*. 2017) and even in EMT (Jefri *et al*. 2015).

Genes differentially expressed within the range of two fold change between D492M and D492HER2 were further classified using PANTHER analysis (Mi *et al*. 2013) in order to establish the biological processes that were enriched in each cell line. Genes more highly expressed in D492HER2 compared to D492M were shown to be involved in the following biological processes: D-aspartate transport, inflammatory response, cell chemotaxis, cellular response to cytokine stimulus, defense response, cellular response to cytokine stimulus, cell proliferation, and organic anion transport, among others (Fig. 2b). Interestingly, YKL-40 was found enriched in four of the 18 groups, as highlighted in yellow in Fig. 2b.

Complementing the gene expression data, we also measured by mass spectrometry the secretion of the proteins by D492M and D492HER2. Analysis of the total secreted proteins in the conditioned media (CM) from D492M and D492HER2 revealed YKL-40 was found in much higher concentration in CM from D492HER2 (97.8 LFQ intensity) than D492M (5.4 LFQ intensity) (Fig. 2*c*). After these promising results, we strengthened our focus on YKL-40 as a firm candidate that could lead to differences between D492M and D492HER2.

### Knockdown of YKL-40 in D492HER2 inhibits migration and invasion

**Figure 3.**
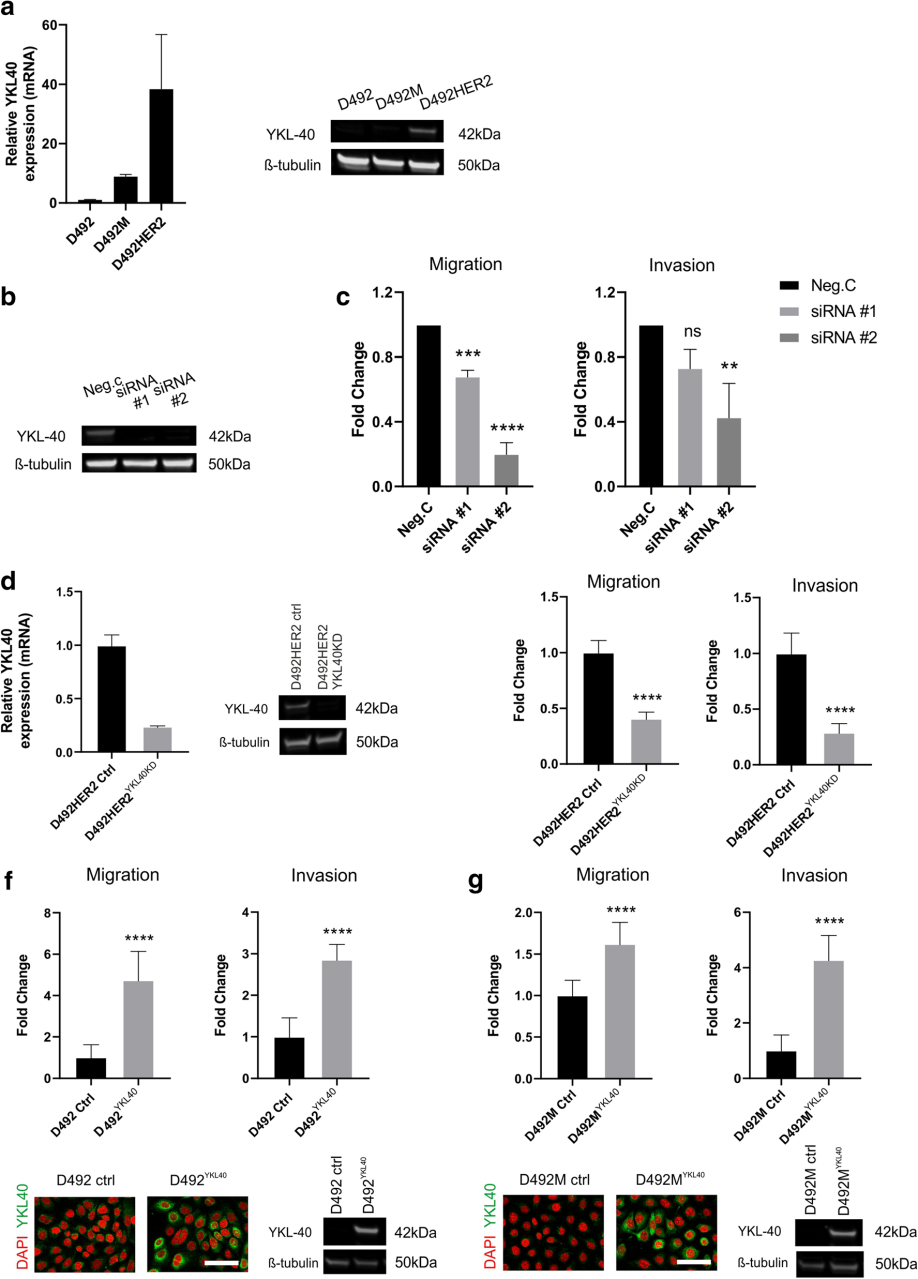
YKL-40 affects migration and invasion in D492 cell lines. *a* YKL-40 is more highly expressed in D492HER2, compared to D492 and D492M, both at mRNA and protein level. *b*, *c* Transient siRNA knockdown of YKL40 reduced the ability of D492HER2 to migrate and invade in vitro (mean ± SD, *n* = 3). Statistical significance was tested using one-way analysis of variance (ANOVA) and Dunnett’s multiple comparison test (**p ≤ 0.01, ***p ≤ 0.001, ****p ≤ 0.0001). *d*, *e* Knock-down of YKL-40 in D492HER2 in stable cell lines generated by CRISPR/Cas9 technology reduced the ability of D492HER2 to migrate and invade similarly to transient transfection. *f*, *g* Accordingly, overexpression of YKL40 in D492 and D492M using CRISPRa system increased their ability to migrate and invade. Overexpression of YKL-40 was confirmed by immunofluorescence staining (*Scale bar* = 100 μm) and western blotting. Migration and invasion assay results are shown as average of eight replicates (mean ± SD). Unpaired *t* test was used to test significance (**p ≤0.01, ***p ≤ 0.001, ****p ≤ 0.0001).

To explore the functional role of YKL-40 in D492HER2, we did both transient and stable knockdown (KD). First, we confirmed the expression of YKL-40 at mRNA and protein level in D492, D492M, and D492HER2 demonstrating its abundant expression in D492HER2 (Fig. 3*a*). Transient knockdown with two different siRNAs greatly reduced YKL-40 expression in D492HER2 cells (Fig. 3*b*) and resulted in a significant decrease in migration and invasion (Fig. 3*c*). To further corroborate these effects, we generated stable YKL-40 KD using CRISPR technology in D492HER2 cells (Fig. 3*d*). Similar to transient KD of YKL-40, the stable KD cell line showed a reduction in migration and invasion compared to control D492HER2 cells (Fig. 3*e*). To analyze if overexpression of YKL-40 in D492 and D492M resulted in increased migration and invasion, we stably overexpressed YKL-40 in these cells and, indeed, this resulted in a large increase in both migration and invasion (Fig. 3*f*, *g*). No differences were seen with regard to cell proliferation regardless if YKL-40 was knocked down in D492HER2 or overexpressed in D492/D492M (data not shown). Collectively, these results demonstrate that in our cell system, YKL-40 increases migration and invasion.

### YKL-40 expression in D492HER2 is linked to vascular network formation

**Figure 4.**
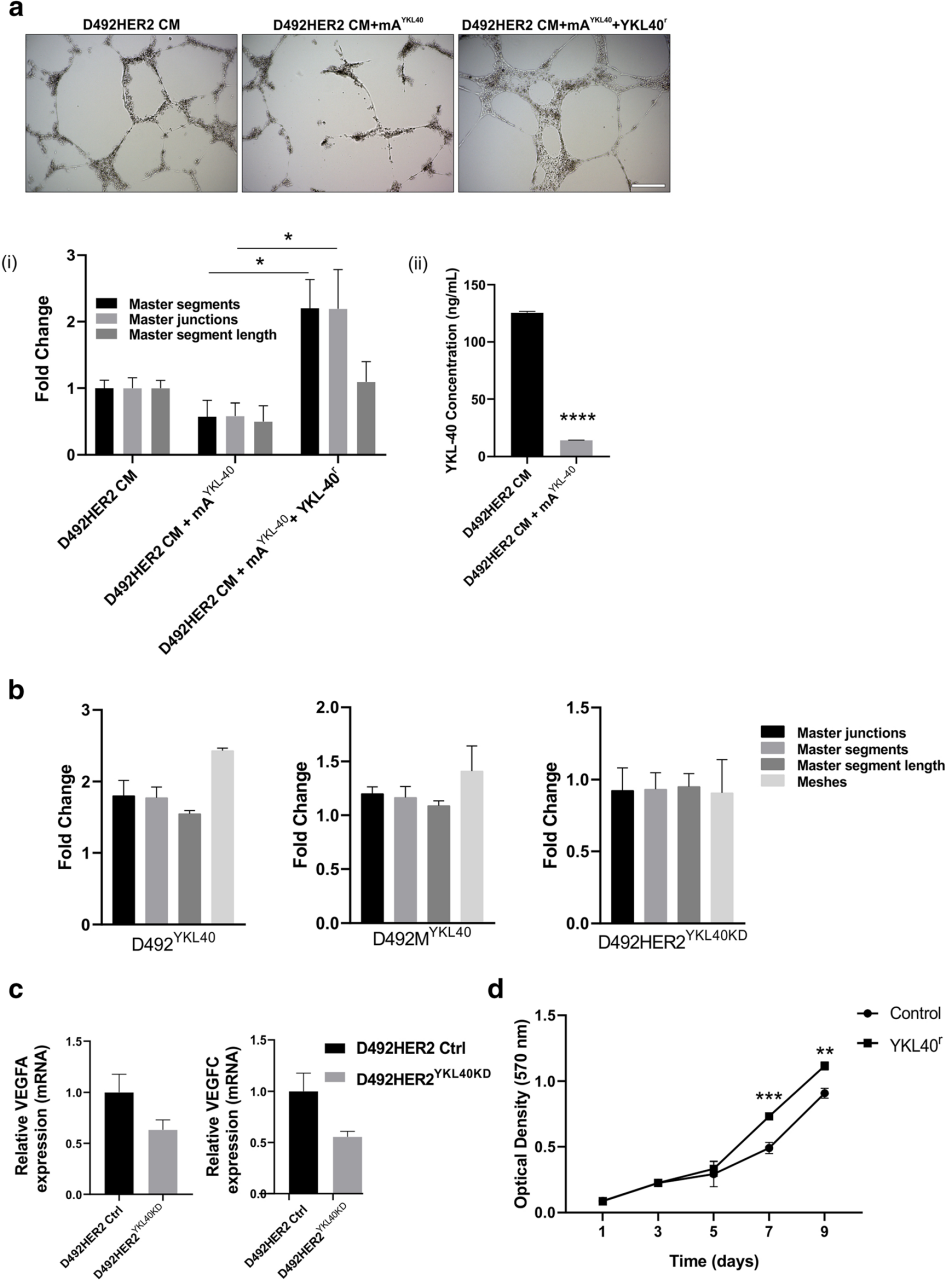
YKL-40 expression in D492HER2 is linked to increased potential to stimulate angiogenesis. (*a*) Conditioned media (CM) from D492HER2 cells stimulates angiogenesis in an in vitro angiogenesis assay. This effect was abrogated upon YKL-40 neutralization by a monoclonal antibody (mA^YKL40^) but rescued by the addition of recombinant YKL-40 protein (YKL40^r^) (*Scalebar* = 200 μm). (i) Parameters of angiogenesis were measured and analyzed by ImageJ angiogenesis analyzer plug (mean ± SD) and significance was assessed with one-way analysis of variance (ANOVA) and Tukey’s multiple comparison test (*p ≤ 0.05). (ii) Decreased concentration of YKL-40 upon incubation of cells with mA^YKL40^ was verified with ELISA. Significance was tested with an unpaired t-test (****p ≤ 0.0001). (*b*) When YKL-40 was overexpressed in D492 and D492M, there was an increase in the capillary network formation, whereas stable knockdown of YKL-40 in D492HER2 showed a tendency to reduced capillary network formation. (*c*) Knockdown of YKL-40 reduced the expression of other pro-angiogenic inducers, VEGFA and VEGFC. (*d*) Finally, recombinant YKL-40 protein (YKL-40^r^) induced proliferation of endothelial cells when added to the media. Unpaired *t* test was used to test significance (**p ≤ 0.01, ***p ≤ 0.001).

YKL-40 has previously been linked to angiogenesis (Shao *et al*. 2009, 2011). In order to see if increased expression of YKL-40 in D492HER2 was linked to increased potential to stimulate angiogenesis in an in vitro assay, we treated HUVECs on top of Matrigel with CM from D492HER2 with and without a functional blocking antibody against YKL-40 (mA^YKL40^). Blocking YKL-40 directly reduced the ability of CM medium to induce HUVECs to form capillary-like networks. This blocking effect was reversed by adding recombinant YKL-40 protein (YKL-40^r^) (Fig. 4*a*).

In agreement, CM from D492 and D492M stably overexpressing YKL-40 increased HUVEC network formation on top of Matrigel, whereas stable knockdown of YKL-40 in D492HER2 cells reduced HUVECs network formation (Fig. 4*b*). Additionally, the reduction of YKL-40 in the stable cell line revealed reduced expression of other pro-angiogenic genes, like VEGF-A and VEGF-C (Fig. 4*c*), suggesting a synergic pro-angiogenic effect induced in D492HER2. Furthermore, adding recombinant YKL-40 protein (YKL-40^r^) to media not only stimulated tube formation on endothelial cells, but also increased proliferation rates of HUVECs (Fig. 4d).

### YKL-40 affects phenotype of D492 cell lines when cultured in 3D culture

When YKL-40 was downregulated in D492HER2 by transient or stable transfection, no phenotypic changes were seen in monolayer culture and EMT markers in immunostaining and western blot did not show any differences; thus, the EMT phenotype remained unaltered in 2D. Due to the transient nature of using siRNA, it was difficult to explore if there were any phenotypic changes occurring in 3D culture. In contrast, 3D culturing of cells with stable knockdown of YKL-40 revealed differences in phenotype of the D492 cell lines.

**Figure 5.**
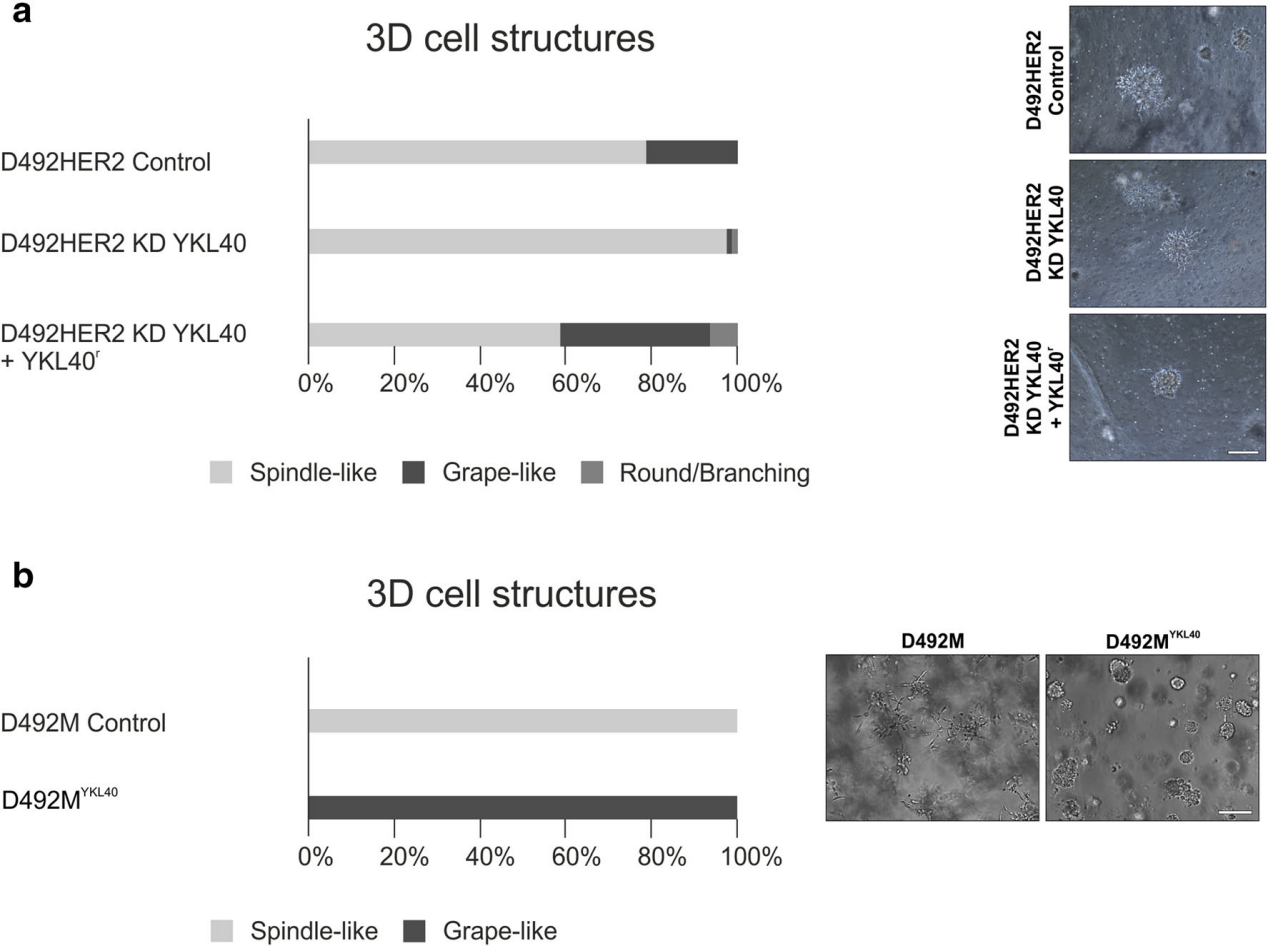
YKL-40 affects the phenotype of D492 cell lines in 3D. *a* CRISPR/Cas9-mediated knockdown of YKL-40 in D492HER2 cells, increased the number of spindle-like structures in a 3D culture, but grape-like structures were recovered with addition of recombinant YKL-40 protein (YKL40^r^) to the media (*Scalebar* = 200 μm). *b* Overexpression of YKL-40 in D492M shifted the phenotype from spindle-like structures to grape-like structures in 3D (Scalebar = 200 μm).

In 3D culture, D492HER2 formed two colony shapes, spindle-like and grape-like (Ingthorsson *et al*. 2015). Interestingly, when YKL-40 was knocked down, there was a dramatic change in the proportion of spindle-like and grape-like structures, with the proportion of grape-like structures being reduced from 21.4% to 1% (Fig. 5*a*). This subsequent change in the 3D phenotype of D492HER2 cells with knockdown of YKL-40 was similar to D492M cells that only showed a spindle-like phenotype (Sigurdsson *et al*. 2011). However, when the recombinant YKL-40 protein (YKL-40^r^) was added to the media of D492HER2 with YKL-40 KD, the grape-like structures were rescued to 32.8% of the total 3D structures (Fig. 5*a*).

To confirm the shift to grape-like structures with the addition of YKL-40, D492M cells overexpressing YKL-40 were seeded in 3D cell culture. Remarkably, the entire cell population lost the ability to form spindle-like structures, and instead only grape-like structures were observed (Fig. 5*b*).

## Discussion

In this study, we compared phenotypic and functional differences between two isogenic cell lines that share a partial-EMT phenotype but differ in terms of oncogenic properties. D492M is non-tumorigenic, whereas D492HER2 is tumorigenic. We identified YKL-40, also known as a chitinase-3-like protein 1 (CHI3L1), as a protein highly expressed and secreted in D492HER2 compared to D492M. Functional in vitro studies demonstrated that YKL-40 is involved in migration and invasion of D492HER2. Furthermore, blocking YKL-40 in conditioned media (CM) from D492HER2 reduced the ability of the D492HER2-derived CM to induce formation of capillary like network in an in vitro angiogenesis assay, which conversely can be rescued with the addition of recombinant YKL-40 protein. Finally, KD of YKL-40 in D492HER2 changed the ratio of spindle- and grape-like structures in favor of spindle-like structures, which could be reverted when the recombinant protein was added to the KD cells in 3D culture.

The term EMT is used for describing phenotypic changes where epithelial cells lose and gain epithelial and mesenchymal traits, respectively. Very rarely, a complete EMT occurs, and rather intermediate states are the most frequent phenotypes. Thus, it is common that traces of epithelial traits remain and a full mesenchymal phenotype is often incomplete resulting in a large spectrum of EMT phenotypes (Nieto 2013).

D492M and D492HER2 share some EMT properties such as loss of E-cadherin and cytokeratins and formation of a spindle-shaped phenotype in monolayer. D492M has a more fixed EMT phenotype with low or absent expression of epithelial markers, including microRNAs, and gain of mesenchymal markers (Sigurdsson *et al*. 2011; Hilmarsdottir *et al*. 2015). In a recent paper, we demonstrated that reintroduction of miR-200c-141 into D492M was sufficient to revert the phenotype to epithelial lineage, albeit only to luminal epithelial cells. Further studies demonstrated that the basal cell transcription factor P63 was necessary to recover the basal/myoepithelial phenotype. Co-transfection of miR-200c-141 and P63 into D492M was sufficient to regain the original phenotype of D492 (Hilmarsdottir *et al*. 2015). On the other hand, D492HER2 shows more intermediate EMT phenotype than D492M and could be defined as a cell line with partial-EMT (Ingthorsson *et al*. 2015). Even though D492HER2 migrates, invades, and proliferates more, it is more susceptible to apoptosis, its glucose metabolism is more similar to the one in the epithelial cell line (D492), and the expression levels of the regulatory epithelial miRNAs are reduced but not as dramatic as in the case of D492M. Moreover, there are evident phenotypic differences between these two cell lines when cultured in 3D in reconstituted basement membrane matrix, as D492M forms exclusively spindle-shaped EMT-like colonies in contrast to D492HER2 that forms mixture of spindle-shape and grape-like colonies.

Triple-negative breast cancer cell lines are often spindle-shaped with more prominent EMT phenotype in 3D rBM while cell lines with high HER2 expression are associated with grape-like phenotype (Han *et al*. 2010). We have previously shown that D492M and D492HER2 form spindle-shaped and grape-like structures in 3D rBM, respectively (Sigurdsson *et al*. 2011; Ingthorsson *et al*. 2016). That overexpression of YKL-40 in D492M shifts the phenotype toward grape-like structures may indicate that YKL-40 confers increased plasticity to D492M. Further studies in our laboratory aim to unravel whether YKL-40 expression is associated with tumorigenic potential of D492M and D492HER2.

Comparing and analyzing gene-wide expression and protein-wide secretion of D492M and D492HER2, YKL-40 was the most relevant candidate to study the differences between the cell lines. YKL-40 is a secreted glycoprotein of a still unclear function. The protein belongs to the chitinase family, but it lacks hydrolase activity (Renkema *et al*. 1998). It is expressed by several cell types including macrophages, neutrophils, epithelial cells, synovial cells, chondrocytes, smooth muscle cells, and some cancer cells (Recklies *et al*. 2002; Johansen *et al*. 2006). It is suggested to be involved in inflammation and angiogenesis, as well as survival and growth in tumors (Johansen *et al*. 2006). It is highly secreted in inflammation diseases (Roslind and Johansen 2009; Bonneh-Barkay *et al*. 2010) and some cancers, such as breast cancer, melanoma, glioblastoma, or small cell lung carcinoma (Johansen *et al*. 2006; Shao *et al*. 2011; Jefri *et al*. 2015; Libreros and Iragavarapu-Charyulu 2015). Recently, it has been linked to idiopathic pulmonary fibrosis (Zhou *et al*. 2014) and immunosuppressive effects by cancer-associated fibroblasts in breast cancer (Cohen *et al*. 2017).

We have confirmed here that changes in intrinsic expression of YKL-40 in D492HER2 lead to differences in migration and invasion behavior of the cells. When YKL-40 is downregulated by transient or stable knockdown, D492HER2 becomes less migrative and invasive. Migration and invasion are important processes that support cancer development and progression (Hanahan and Weinberg 2011) what indicates that YKL-40 may play a key role in invasiveness and dissemination of cancer cells.

YKL-40 has been previously linked to EMT in non-small cell lung cancer (Jefri *et al*. 2015), yet in our study, when YKL-40 was downregulated in D492HER2, its EMT phenotype in monolayer remained unaltered and there were no differences in expression of EMT markers. Nevertheless, high expression of YKL-40 or the addition of the recombinant protein has been related to an increase in the grape-shaped colonies in 3D cell cultures of the EMT-derived cell lines. This fact suggests that YKL-40 may have been involved in the plasticity of the cells, as the phenotype in 3D was changed when its expression was reduced in D492HER2 and then rescued when the recombinant protein was added. This is also interesting, since the grape-like structures are often associated with HER2-expressing cell lines (Kenny *et al*. 2007).

A number of papers have shown association between YKL-40 and HER2 expression in breast cancer. Wang *et al*. showed by meta-analysis of existing databases that YKL-40 is associated with poor prognosis in breast cancer patients (Wan *et al*. 2017). Kang *et al*. have demonstrated that YKL-40 expression in breast cancer is associated with Her-2 and basal-like molecular subtype of breast cancer (Kang *et al*. 2014). Also, Shao *et al*. showed an association between YKL-40 and Her2 subtype (Shao *et al*. 2011). Although, the association between YKL-40 and HER2 has been demonstrated, it is, however, to our best knowledge, still not clear if there are any direct or indirect molecular interactions between these two proteins in breast cancer. Using the breast mark database (Madden *et al*. 2013), we were however not able to link high or low expression of CHI3L1 in distinct subtype of breast cancer to increased or reduced survival.

PANTHER classification of the differentially regulated genes showed that YKL-40 is in enriched in biological processes related to interactions with the microenvironment that can support the development of tumors by cancer cells (inflammatory response, cell chemotaxis, cellular response to cytokine stimulus, and defense response). We demonstrate here that YKL-40 interacts with the microenvironment and more concretely is contributing to the angiogenesis-inducing effect of D492HER2.

We have demonstrated that YKL-40 secreted by D492HER2 has a role in angiogenesis. When YKL-40 protein is reduced in the media by a specific YKL-40 blocking antibody or stable knockdown in D492HER2, angiogenesis is decreased. Conversely, increased levels of YKL-40 in the CM by the addition of recombinant YKL-40 protein or secreted by the cell line reverted the effects to standard tube formation parameters.

Interestingly, the reduction of YKL-40 in D492HER2 by the stable knockdown cell line revealed the reduction of the expression of other pro-angiogenic genes, including VEGF-A and VEGF-C. These genes encode protein ligands that bind and activate VEGF receptor 2 (VEGFR2, Flk-1/KDR), one of the main receptors implicated in the induction of angiogenesis in endothelial cells (Carmeliet and Jain 2000; Harper and Bates 2008). Indeed, it has been shown that a monoclonal antibody against YKL-40 abolishes YKL-40-induced activation of the membrane VEGF receptor 2 and intracellular signaling mitogen-activated protein (MAP) kinase extracellular signal-regulated kinase (Erk) 1 and Erk 2 (Faibish *et al*. 2011).

However, the role of YKL-40 in cancer progression may not be exclusively derived from cancer cells and not limited to promote interactions with endothelial cells. Recently, it has been described that YKL-40 is highly secreted by fibroblasts associated to cancer (CAFs) in breast-promoting tumor growth and facilitating metastasis. Moreover, YKL-40 released by CAFs supports recruitment of macrophages and promotes the switch to tumor-associated macrophages (TAMs) (Cohen *et al*. 2017). Furthermore, TAMs in breast cancer express YKL-40 where the secreted protein increases inflammation and angiogenesis leading to a worse prognosis and conceivably to metastasis (Shao 2013). Collectively, this suggests that YKL-40 has a complex role in malignancy that may provide the signals for further progression involving changes within cancer cells to migrate and invade and interact with cells of the surrounding stroma. Moreover, it also may provide feedback from the microenvironment to support cancer development, tumor growth, and incrementation of dissemination of cancer cells in metastasis.

## Conclusion

In conclusion, our data suggest that YKL-40 may provide D492HER2 with increased aggressiveness, evidenced by enhanced migration and invasion. Furthermore, YKL-40 may also support cancer progression by facilitating angiogenesis and may therefore be of interest as a potential novel therapeutic target in HER2-positive breast cancer.

## Electronic supplementary material

ESM 1 (JPG 337 kb)
