# Supplementary figures and images for: YKL-40/CHI3L1 facilitates migration and invasion in HER2 overexpressing breast epithelial progenitor cells and generates a niche for capillary-like network formation
Source: In Vitro Cell Dev Biol Anim. 2019 Sep 3;55(10):838–53. doi: 10.1007/s11626-019-00403-x (PMC6881255; doi:10.1007/s11626-019-00403-x)

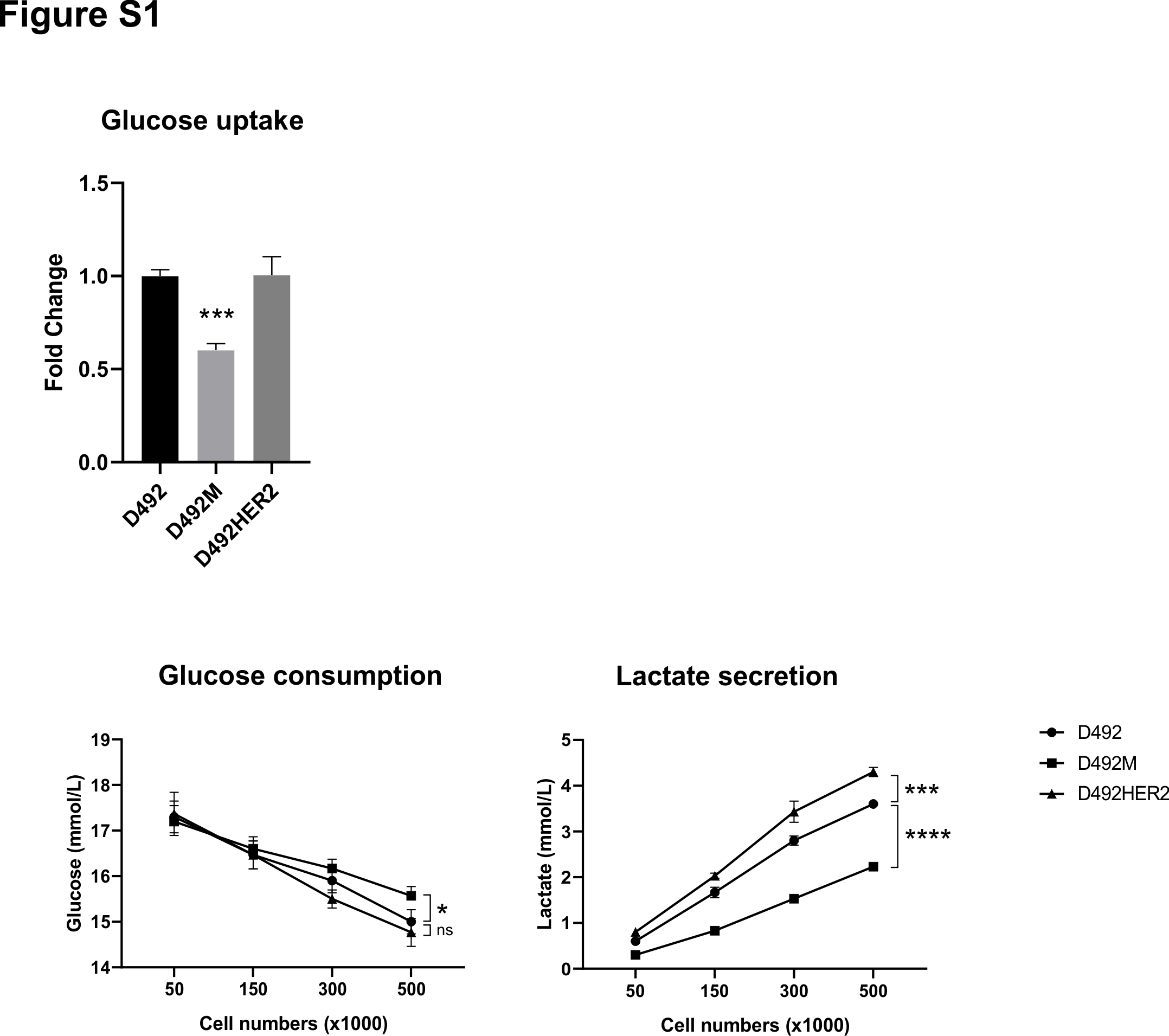

Supplement: Supplementary file 1 — (JPG 337 kb) [file 11626_2019_403_MOESM1_ESM.jpg]
